# Ultrasound-Measured Skin-to-Epiglottis Distance as a Predictor of Difficult Intubation in Obese Patients: A Prospective Observational Study

**DOI:** 10.3390/jcm14062092

**Published:** 2025-03-19

**Authors:** Kazım Ersin Altınsoy, Bahar Uslu Bayhan

**Affiliations:** 1 Department of Emergency Medicine, Gaziantep Islam Science and Technology University, Gaziantep City Hospital, 27470 Gaziantep, Türkiye; 2 Department of Anesthesia and Reanimation, Gaziantep City Hospital, 27470 Gaziantep, Türkiye; dr.b.uslu@gmail.com

**Keywords:** airway management, difficult intubation, obesity, skin-to-epiglottis distance, ultrasound

## Abstract

**Background/Objectives**: Difficult intubation is a significant clinical issue in emergency medicine as well as anesthesia practice, occurring more frequently in obese patients. Traditional assessment methods may not be sufficient to predict difficult intubation. This study aims to evaluate the ability of ultrasound-measured skin-to-epiglottis distance (SED) to predict difficult laryngoscopy in obese patients and investigate its applicability in clinical practice. **Methods**: This prospective observational study was conducted between February 2024 and January 2025 at Gaziantep City Hospital on obese patients undergoing bariatric surgery. Patients aged 18 years and older with an American Society of Anesthesiologists (ASA) classification of I-II-III were included in the study. Demographic data, standard airway assessment parameters (neck circumference, thyromental distance, sternomental distance, etc.), and ultrasound-measured skin-to-epiglottis distance were recorded. All intubation procedures were performed by a single experienced anesthesiologist following standard protocols, and laryngoscope view was assessed according to the Cormack–Lehane classification. **Results**: Among the 61 patients included in the study, 16.4% were classified as having a difficult airway, and 13.1% experienced difficult intubation. No significant correlation was found between standard airway assessment parameters and difficult intubation. However, ultrasound-measured skin-to-epiglottis distance (SED) was significantly higher in patients with difficult intubation (*p* = 0.004), making it a strong predictor. Additionally, modified Mallampati (*p* < 0.001), modified Cormack–Lehane (*p* = 0.003), and Wilson scores (*p* = 0.001) were significant in predicting difficult airway, although Wilson score was not significant for difficult intubation (*p* = 0.099). **Conclusions**: Our study suggests that ultrasound-measured skin-to-epiglottis distance may be a valuable predictor of difficult intubation in obese patients. Given the limitations of preoperative assessment methods, incorporating ultrasound into airway evaluation as a complementary tool provides significant benefits. Larger-scale studies in the future are necessary to further assess the clinical efficacy of this method.

## 1. Introduction

William Morton’s first application of ether anesthesia marked a significant turning point in the history of modern anesthesia [1]. This development not only provided a revolutionary advancement in the field of medicine but also introduced the responsibility and complexity of anesthesia applications. Despite technological advancements and scientific progress, emergency physicians and anesthesiologists continue to face fundamental challenges in airway management. One of these challenges is difficult intubation [2].

There are numerous studies in the literature aimed at predicting difficult intubation, and various clinical tests have been developed for this purpose [3]. Parameters such as thyromental distance, sternomental distance, neck circumference, and the distance between incisors are practical tests that can be applied during pre-anesthetic evaluation. However, none of these have a definitively proven predictive value [4]. Although combining these tests improves diagnostic accuracy, additional methods are still needed [5]. Difficult intubation risk is particularly well known in obese patients, and airway management in this patient group becomes even more complex when using standard predictive tests [6,7]. Due to the anatomical changes induced by obesity, studies have shown that obese patients have higher rates of difficult laryngoscopy and failed intubation. Consequently, the need for additional evaluation methods that can more reliably predict difficult intubation in obese patients has become increasingly evident.

In recent years, ultrasound has been increasingly utilized in clinical practice due to its portability, non-invasiveness, and ease of use [8]. Studies on the use of ultrasound in airway management have drawn attention, and its application as a rapid and reliable assessment tool is becoming more widespread in emergency departments, operating rooms, and intensive care units.

There are studies in the literature suggesting that the measurement of the distance from the skin to the epiglottis (DSE) has the potential to predict difficult intubation [8]. Although DSE is proposed as a good predictor, a universally accepted cutoff value has not yet been established for this parameter. Moreover, the number of studies conducted on specific patient groups, such as obese individuals, remains quite limited. The aim of this study is to evaluate the ability of DSE to predict difficult laryngoscopy and to investigate its applicability in daily clinical practice.

## 2. Materials and Methods

Our study was conducted between February 2024 and January 2025 at Gaziantep City Hospital, focusing on the profile of obese patients undergoing bariatric surgery. Prior to patient enrollment, we conducted a power analysis using G*Power 3.1 (effect size f^2^ = 0.15, α = 0.05, power = 0.80, number of predictors = 5), which determined a required sample size of 43 patients. However, we included 61 patients to enhance statistical power and account for potential dropouts. This was a prospective, observational study conducted in accordance with the Declaration of Helsinki. This study was prospective and observational, and after obtaining ethics committee approval, we ensured that all patients signed the informed consent form before participation.

Patients scheduled for obesity surgery were evaluated in the preoperative preparation room to determine whether they met the inclusion or exclusion criteria. Those found suitable were informed about the study, and the informed consent form was obtained in the same setting. Patients aged 18 years or older with an American Society of Anesthesiologists (ASA) classification of I-II-III, who were scheduled for elective bariatric surgery, were included in the study. Exclusion criteria consisted of patients under 18 years of age, those classified as ASA IV-V, and individuals with congenital facial anomalies, temporomandibular joint (TMJ) ankylosis, facial trauma, or burns. Additionally, patients with cognitive impairment, cervical spine disease, or the presence of a beard were excluded, as well as those who did not sign the informed consent form.

Demographic data, including age, ASA classification, height, body weight, and body mass index (BMI), were recorded for each patient. To assess the risk of a difficult airway, several parameters were documented, including neck circumference (measured in centimeters), neck mobility (<80°/≥80°), thyromental distance ratio, sternomental distance, Wilson risk score, and the modified Mallampati score.

All measurements in centimeters were obtained using a non-stretchable measuring tape to ensure accuracy and reliability.

**Figure 1:**
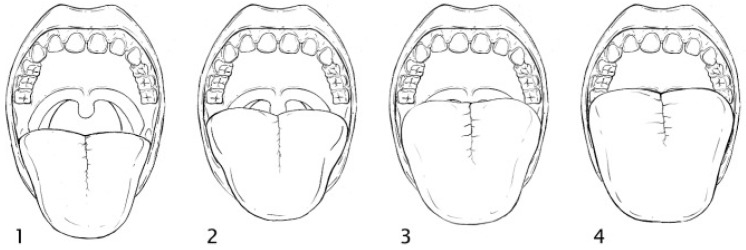
Modified Mallampati scoring system (adopted from [9]). Class 1: faucial/tonsillar pillars, uvula and soft palate are all visible. Class 2: partial visibility of the faucial/tonsillar pillars, uvula and soft palate. Class 3: base of the uvula, soft and hard palate visible. Class 4: only hard palate is visible.

Measurement and Classification Details:

- Thyromental distance ratio (cm):
- Measured from the midpoint of the thyroid cartilage to the chin, in both neutral position and maximum head extension.
- Sternomental distance (cm):
- Measured from the lower border of the mandible to the upper edge of the sternum, with the head in full extension and the mouth closed.
- Airway classification based on oropharyngeal visibility (Modified Mallampati Scoring System) is illustrated in Figure 1, which categorizes patients into four grades.

The risk stratification for airway management is presented in Table 1, which categorizes patients based on weight, jaw movements, mandible position, dental structure, and head-neck mobility.

After recording the necessary data, all patients placed on the operating table were positioned in the ramped position (elevated position) during intubation. This was performed to ensure optimal airway opening and to eliminate variability among patients.

### 2.1. Definition of Difficult Airway and Difficult Intubation

A difficult airway was defined as the presence of anatomical or clinical factors that could complicate face mask ventilation or endotracheal intubation. These included a modified Mallampati score of 3–4, Wilson score ≥ 2, limited neck mobility (<80°), or increased neck circumference.

A difficult intubation was defined as a Cormack–Lehane grade 3–4 view during direct laryngoscopy, requiring multiple intubation attempts or additional airway management aids (e.g., bougie, video laryngoscope). All cases of difficult intubation were evaluated and confirmed by both an experienced anesthesiologist and an emergency physician, each with over 10 years of experience.

### 2.2. Ultrasound Measurements

**Figure 2:**
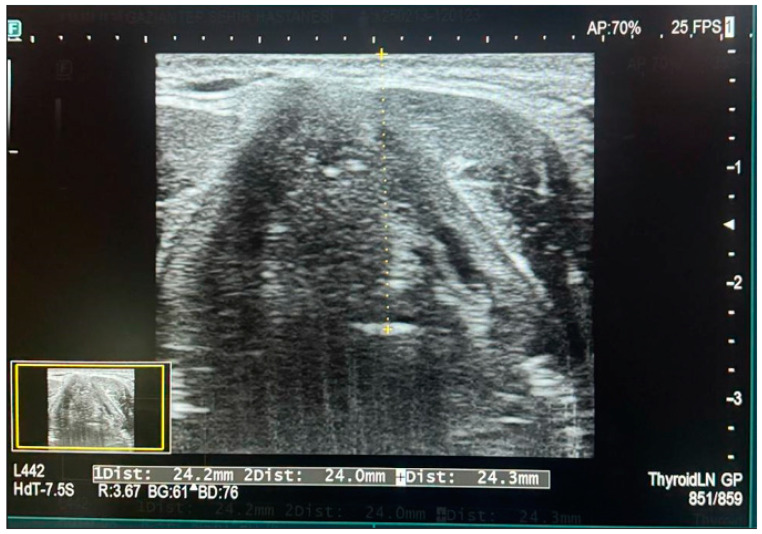
Measurement of skin-to-epiglottis distance via ultrasound.

Ultrasound measurements were performed by the same sonographer each time, using different degrees of cephalad and caudal angulation in the transverse plane. A Sonosite M-Turbo ultrasound device equipped with a 10–13 MHz linear transducer was used for the measurements. The ultrasound probe was positioned at the thyrohyoid membrane level on the neck. The epiglottis was identified as a curved, hypoechoic structure in the transverse plane at the thyrohyoid membrane level. Once all soft tissue structures were anatomically identified, the distance from the skin to the epiglottis (DSE) was measured. For each individual, three separate measurements were taken: one at the midline, one on the right side, and one on the left side of the epiglottis. The average of these three measurements was recorded as the final value (see Figure 2).

**Figure 3:**
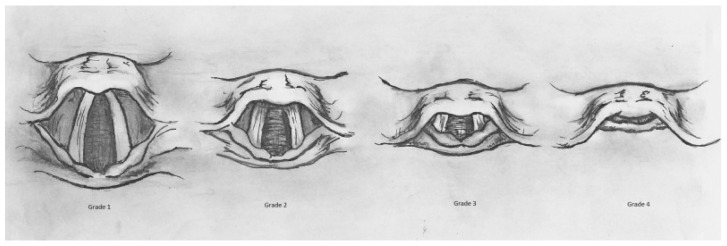
Cormack–Lehane classification.

Before induction, all patients underwent preoxygenation with 100% oxygen for 3 min to ensure adequate oxygen reserves. The difficulty of positive pressure ventilation was assessed during ventilation. For intubation, a Macintosh laryngoscope blade size 3 was used for female patients, while Macintosh blade size 4 was used for male patients. Endotracheal intubation was performed using a 7.0 mm endotracheal tube (ETT) for female patients and an 8.0 mm ETT for male patients, both of which were preloaded with a stylet. To minimize variability, all intubations were performed by a single anesthesiology specialist. The laryngoscopic view was classified using the Cormack–Lehane (C&L) classification by the laryngoscopist, without applying cricoid pressure. The success of direct laryngoscopy and the need for additional intubation aids were also recorded. The use of additional intubation assistive devices was documented as an indication of difficult intubation. The Cormack–Lehane classification (Figure 3, adapted from [10]) was used to assess glottic visualization during direct laryngoscopy. It consists of four grades: Grade 1, where the vocal cords are fully visible; Grade 2, where only the posterior portion of the vocal cords and arytenoids are seen (subdivided into Grade 2a, where the posterior vocal cords are visible, and Grade 2b, where only the arytenoids and posterior epiglottis are seen); Grade 3, where only the epiglottis is visible without the vocal cords; and Grade 4, where neither the vocal cords nor the epiglottis can be seen, indicating the highest difficulty in intubation.

### 2.3. Statistical Analysis

The study data were analyzed using SPSS (Statistical Package for the Social Sciences) version 27.0 and MedCalc version 22.007. Numerical data were expressed as mean ± standard deviation and median (interquartile range, IQR), while frequency data were presented as percentages. For the comparison of categorical variables, the chi-square test was used. The distribution of continuous variables was assessed using the Kolmogorov–Smirnov test. Since the data did not follow normal distribution, non-parametric tests were applied for continuous variables. All hypotheses were tested using a two-tailed approach, and a *p*-value < 0.05 was considered statistically significant.

## 3. Results

After applying the inclusion and exclusion criteria, data from 61 patients were collected. The demographic data and measurements of the patients are presented in Table 2.

Among the study participants, 16.4% were classified as having a difficult airway. Additionally, 13.1% of all patients experienced difficult intubation (Table 3).

Patients were divided into two groups based on difficulty of intubation. The only significantly different parameter between the two groups was the skin-to-epiglottis distance (DSE) measured by ultrasound (USG), with a *p*-value of 0.004 (Table 4).

Patients were divided into two groups according to difficult airway status. Among the measured parameters, BMI and sternomental distance were found to be statistically significantly different between the two groups (Table 5).

Our patient group represents a specialized population. Therefore, we aimed to evaluate the most commonly used scoring systems—modified Mallampati, modified Cormack–Lehane, and Wilson score—within our own patient cohort in terms of difficult airway and difficult intubation. For modified Mallampati and modified Cormack–Lehane scores, patients with scores of one or two were classified as having easy intubation, while those with scores of three or four were categorized as having difficult intubation. Similarly, for the Wilson score, a score of ≥2 was considered an indicator of difficult intubation. The modified Mallampati score was found to be statistically significant for predicting difficult airway (Table 6). Meanwhile, all three parameters—modified Mallampati, modified Cormack–Lehane, and Wilson score—were statistically significant predictors of difficult intubation (Table 7).

Table 8 presents the results of ROC analysis for the measurements of skin-epiglottic distance, neck circumference, modified Mallampati, modified Cormack–Lehane, and Wilson score obtained by ultrasound assessment.

**Figure 4:**
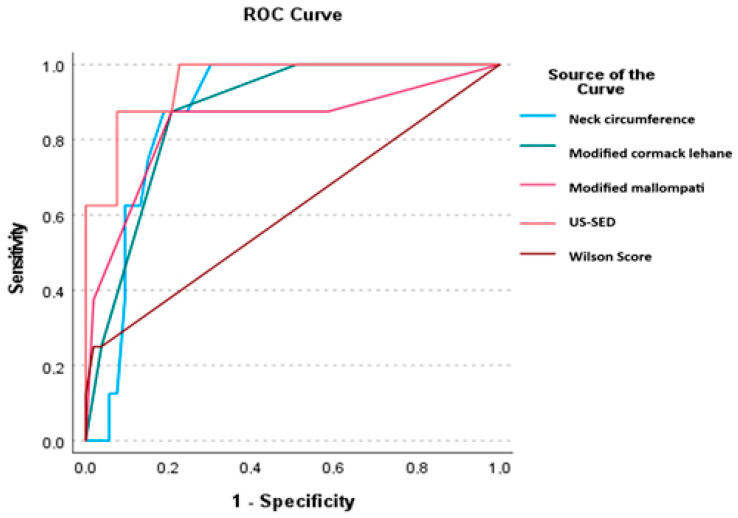
ROC analysis of skin-to-epiglottis distance and neck circumference. ROC analysis was performed. The cutoff value, sensitivity, and specificity were calculated using MedCalc. US-SED: ultrasound with skin-epiglottis distance.

In the ROC analysis, the sensitivity and specificity of the skin-to-epiglottis distance in predicting difficult laryngoscopy were found to be 82.5% and 79.45%, respectively, indicating a high predictive value. In comparison, sensitivity was 75.79% and specificity was 60.96% for neck circumference, and sensitivity was 86.5% and specificity was 78.5% for modified Mallampati (Table 8 and Figure 4).

To identify independent predictors of difficult intubation, a multivariate logistic regression analysis was performed in our study. The variables included in the analysis were the modified Mallampati score, modified Cormack–Lehane classification, neck circumference, Wilson risk score, and the skin-to-epiglottis distance measured via ultrasound. According to the analysis results, the *p*-value for the constant term and skin-to-epiglottis distance was found to be <0.001, indicating that this variable is a strong independent predictor of difficult intubation (Table 9). This finding suggests that skin-to-epiglottis distance may serve as a significant parameter in airway assessment and should be considered in clinical decision-making processes.

## 4. Discussion

Difficult airway management is a significant clinical challenge in emergency, anesthesia, and intensive care units, especially in obese patients, where intubation difficulties are more common [6]. In our study, we evaluated the importance of skin-epiglottic distance measured by ultrasound (USG) in predicting difficult induction in obese patients. The selection of neck circumference and skin-epiglottic distance (SED) as parameters in our ROC analysis is based on their widespread discussion in the literature as potential predictors of difficult intubation in obese patients. Neck circumference is considered an important parameter due to its potential effects on airway anatomy, and previous studies have demonstrated a strong association with difficult intubation [5,11]. As a result, neck circumference was included as a key assessment parameter in our study. It was measured at the level of the cricoid cartilage using a non-flexible measuring tape, ensuring contact with the skin but without compression. Two measurements were taken, and the average was recorded for accuracy.

Conversely, skin-epiglottic distance (SED) is a relatively new parameter assessed via ultrasound, reflecting airway depth in obese individuals. The ability of SED to visualize airway structures makes it a promising tool, particularly in cases where traditional assessments may be less reliable [8]. Given this potential, we incorporated SED into our ROC analysis to evaluate its predictive value for difficult intubation. Both parameters were analyzed as predictors of difficult intubation and assessed for their clinical relevance.

Our findings suggest that skin-epiglottic distance may be a statistically significant predictor of difficult intubation. Similarly, BMI and sternomental distance showed significant correlations with difficult airway. However, other traditional anthropometric measurements such as height, weight, neck circumference, and thyromental distance did not show significant correlations with difficult intubation or difficult airway.

The existing literature suggests that the relationship between obesity and difficult airway/intubation is multifactorial and that traditional physical assessment methods do not always provide reliable predictive value [4,11,12]. Consistent with these findings, our study found significant correlations among traditional parameters only for BMI and sternomental distance in difficult airway, whereas the skin-epiglottic distance measured by USG was significantly associated with difficult intubation. A review of meta-analysis studies in the literature suggests that ultrasound-based methods, such as measuring skin-to-epiglottis distance, have shown higher sensitivity and specificity [13]. This result indicates that ultrasound may serve as a valuable tool for airway assessment in difficult airway management. Several previous studies have explored the role of ultrasound in airway evaluation. Studies by Sumidtra et al. have similarly reported that ultrasound-based evaluation of the epiglottis and upper airway structures can help predict difficult intubation [14,15,16].

Modified Mallampati, modified Cormack–Lehane, and Wilson scores were statistically significant in predicting difficult airway. This result suggests that while these scoring systems remain valuable for difficult airway prediction, they may not be sufficient on their own. The Mallampati score, which assesses airway patency based on tongue and oropharyngeal structures, has previously been described as a reliable predictor of difficult airway in several studies [5,11,17]. The modified Cormack–Lehane score, which evaluates glottic structures during laryngoscopy, was also significantly associated with difficult intubation in our study, further supporting its clinical relevance in airway management.

The challenges in airway management in obese patients are influenced by multiple factors, including altered airway anatomy, increased soft tissue mass, fat deposition around the neck, and reduced neck mobility [18,19]. In our study, 18% of patients with restricted neck mobility experienced difficult intubation; however, this association was not found to be statistically significant. While previous studies have reported a correlation between reduced neck mobility and difficult intubation in obese patients, our findings did not confirm this relationship [19]. One possible explanation for this discrepancy is that our sample size may not have been large enough to fully assess the impact of neck mobility on difficult intubation.

On the other hand, the finding that skin-to-epiglottis distance measured via USG was a significant predictor of difficult intubation suggests that ultrasound may play an increasingly important role in airway management. Combining traditional scoring systems with ultrasound measurements could potentially enhance the accuracy of difficult airway prediction [20,21]. Future studies with larger patient cohorts are needed to further investigate the role of ultrasound in airway assessment and its potential integration into routine clinical practice.

This study has several limitations. One of the primary limitations of this study is that all intubations and ultrasound measurements were performed by a single anesthesiologist. While having a single operator conduct all evaluations ensures methodological consistency, it may also introduce operator bias (observer bias). However, it is important to note that if ultrasound measurements were performed by different observers, variability between measurements could arise, potentially compromising the reliability of the method.

Furthermore, this study has several additional limitations. The patient population was selected based on specific inclusion and exclusion criteria, limiting the generalizability of the findings. Expanding the study to include larger, more diverse patient groups could enhance the reliability of the results. Moreover, intubations were carried out by an experienced anesthesiologist, making it difficult to assess the impact of operator-dependent factors, such as experience level and technical differences, on the outcomes. Finally, variations in ultrasound devices used in future studies could lead to differences in results between practitioners, affecting the consistency and reproducibility of the findings.

The integration of ultrasound into routine airway assessment faces several practical challenges. Limitations in ultrasound use have been identified in the literature, particularly regarding the need for specialized training, access to equipment, and time constraints in emergency situations. These barriers may hinder the widespread adoption of ultrasound as a standard tool for airway evaluation in clinical settings. However, advancements in ultrasound technology and the increasing portability of devices may facilitate broader use in the future.

## 5. Conclusions

Our study demonstrated that skin-to-epiglottis distance measured via ultrasound may serve as a significant predictor of difficult intubation in obese patients. The lack of a significant correlation between traditional anthropometric measurements and difficult airway/intubation suggests that clinical predictions based solely on physical examination may not always be reliable. The integration of ultrasound into airway assessment as a complementary approach to traditional methods should be further explored through future research and clinical validation.

## Figures and Tables

**Table 1:** Wilson risk score.

Risk Factors	Risk Level
Weight (kg)	
<90	0
90–110	1
>110	2
Jaw Movements	
Distance between incisors > 5 cm or Subluxation > 0	0
Distance between incisors < 5 cm or Subluxation = 0	1
Distance between incisors < 5 cm or Subluxation < 0	2
Retruded Mandible	
Normal	0
Moderate	1
Severe	2
Protruding Teeth	
Normal	0
Moderate	1
Severe	2
Head and Neck Movements (Degrees)	
>90	0
−90	1
<90	2
Total Score	0–10

cm = centimeters, kg = kilograms.

**Table 2:** Demographic characteristics and measurement data of study patients.

Variable	Mean ± SD; N (%)
Age	34.83 ± 11.22
Gender	
Male	10 (16.4%)
Female	51 (83.6%)
Height (cm)	161.28 ± 8.52
Weight (kg)	121.67 ± 20.23
BMI	46.64 ± 6.37
Neck Circumference (cm)	43.30 ± 6.09
Sternomental Distance (cm)	15.61 ± 2.05
Thyromental Distance (cm)	7.97 ± 1.73

SD = standard deviation, N = number, BMI = body mass index, cm = centimeters, kg = kilograms.

**Table 3:** Airway assessment scores, ASA classification, and intubation difficulty in study patients.

Variable	N (%)
Modified Mallampati score	
Class 1	23 (37.7%)
Class 2	20 (32.79%)
Class 3	14 (22.95%)
Class 4	4 (6.56%)
Modified Cormack–Lehane score	
Class 1	26 (42.62%)
Class 2	17 (27.87%)
Class 3	14 (22.95%)
Class 4	4 (6.56%)
Wilson score	
<2	-
≥2	61 (100%)
Neck mobility	
<80°	11 (18%)
≥80°	50 (82%)
ASA classification	
Class 1	-
Class 2	33 (54.1%)
Class 3	28 (45.9%)
Difficult airway	
Yes	10 (16.4%)
No	51 (83.6%)
Difficult intubation	
Yes	8 (13.1%)
No	53 (86.9%)

SD = standard deviation, N = number, BMI = body mass index, cm = centimeters, ASA = American Society of Anesthesiologists.

**Table 4:** Comparison of parameters between patients with and without difficult intubation.

Parameters	Mean ± *SD*		
	Absent Difficult Airway	Present Difficult Airway	*p* Value *
Height (cm)	161.9608 ± 8.5720	157.8000 ± 9.49620	0.518
Weight (kg)	118.101 ± 19.180	136.1000 ± 2 0.027	0.768
BMI	45.1039 ± 4.345	54.4700 ± 6.4172	0.550
Neck circumference (cm)	45.1039 ± 5.2389	51.1000 ± 4.6780	0.512
Thyromental distance (cm)	8.2647 ± 1.2378	6.500 ± 1.3876	0.650
Sternomental distance (cm)	16.0490 ± 1.8245	13.089 ± 1.6634	0.289
Skin-to-epiglottis distance (USG, mm)	23.0588 ± 2.3590	24.4600 ± 3.7765	0.004 *

* = Independent samples *t*-test was used. A *p*-value < 0.05 was considered statistically significant. mm: millimeter, cm: centimeter, kg = kilogram, BMI= body mass index, USG = ultrasonography.

**Table 5:** Relationship between difficult airway and measurement values.

Parameter	No Difficult Airway (Mean ± SD)	Difficult Airway (Mean ± SD)	*p*-Value *
Height (cm)	161.79 ± 8.40	157.88 ± 9.60	0.535
Weight (kg)	120.42 ± 20.71	130.00 ± 15.77	0.294
BMI	45.82 ± 6.39	52.06 ± 2.60	**0.022**
Neck circumference (cm)	42.30 ± 5.75	49.88 ± 2.99	0.066
Thyromental distance (cm)	8.19 ± 1.74	6.56 ± 1.86	0.285
Sternomental distance (cm)	15.87 ± 1.99	13.88 ± 1.55	**0.032**
Skin-to-epiglottis distance (USG, mm)	22.62 ± 1.85	27.71 ± 2.47	0.119

* = Independent samples *t*-test was used. A *p*-value < 0.05 was considered statistically significant. mm: millimeter, cm: centimeter, kg = kilogram, BMI = body mass index, USG = ultrasonography.

**Table 6:** Relationship between difficult airway and scoring systems.

Parameter	Difficult Airway Present	Difficult Airway Absent	Total	*p*-Value *
Modified Mallampati score				
Easy (1/2)	2	41	43	**<0.001**
Difficult (3/4)	8	10	18	
Modified Cormack–Lehane score				
Easy (1/2)	3	40	43	**0.02**
Difficult (3/4)	7	11	18	
Wilson score				
Easy (<2)	6	51	58	**0.023**
Difficult (≥2)	4	0	3	
ASA score				
Class 2	3	30	33	0.094
Class 3	7	21	28	

* = Chi-square test was used. A *p*-value < 0.05 was considered statistically significant. ASA = American Association of Anesthesiology.

**Table 7:** Relationship between difficult intubation and scoring systems.

Parameter	Difficult Intubation Present	Difficult Intubation Absent	Total	*p*-Value *
Modified Mallampati score				
Easy (1/2)	1	42	43	**<0.001**
Difficult (3/4)	7	11	18	
Modified Cormack–Lehane score				
Easy (1/2)	1	42	43	**<0.001**
Difficult (3/4)	7	11	18	
Wilson score				
Easy (<2)	6	51	57	**0.009**
Difficult (≥2)	2	2	4	
ASA score				
Class 2	4	29	33	0.083
Class 3	4	24	28	

* = Chi-square test was used. A *p*-value < 0.05 was considered statistically significant. ASA = American Association of Anesthesiology.

**Table 8:** ROC analysis of skin-to-epiglottis distance and neck circumference.

Parameter	AUC *	*p*-Value	Sensitivity (%)	Specificity (%)	95% CI (Lower—Upper Limit)
Skin-ppiglottis distance	0.954	<0.001	82.5	79.45	3.00742–7.17607
Neck circumference	0.875	0.01	75.79	60.96	6.4075–11.7435
Modified Mallampati	0.841	0.02	86.5%	78.5%	0.661–1.000
Modified Cormack–Lehane	0.874	0.01	74.8%	64.6%	0.774–0.974
Wilson score	0.610	0.02	75.1%	61.4%	0.373–0.846

* = Area under the curve, CI = confidence interval.

**Table 9:** Multivariate logistic regression analysis.

Parameters	b	SE	95% GA Alt Limit–Üst Limit	t	*p*
Constant	3.964	0.395	3.173–4.755	10.039	<0.001
Modified Cormack–Lehane	−0.046	0.48	−0.143–−0.050	−960	0.341
Modified Mallampati	−0.029	0.53	−0.135–0.077	−545	0.588
Skin-to-epiglottis distance	−0.72	0.14	−0.100–−0.043	−5.053	<0.001
Wilson score	−0.005	0.073	−0.150–0.140	−0.071	0.944
Neck circumference	−0.006	0.008	−0.022–0.009	−0.814	0.419
R2	0.52				

## Data Availability

The datasets generated and analyzed during the current study are available from the corresponding author upon reasonable request.

## Abbreviations

The following abbreviations are used in this manuscript:

ASA	American Society of Anesthesiologists
BMI	Body mass index
C&L	Cormack–Lehane (classification)
CI	Confidence Interval
DSE	Distance from skin to epiglottis
ETT	Endotracheal Tube
IQR	Interquartile range
LD	Linear Dichroism
ROC	Receiver Operation Characteristics
SD	Standard deviation
SPSS	Statistical package for the social sciences
USG	Ultrasonography
